# Autism spectrum disorders and fetal hypoxia in a population-based cohort: Accounting for missing exposures via Estimation-Maximization algorithm

**DOI:** 10.1186/1471-2288-11-2

**Published:** 2011-01-05

**Authors:** Igor Burstyn, Xiaoming Wang, Yutaka Yasui, Fortune Sithole, Lonnie Zwaigenbaum

**Affiliations:** 1 Department of Medicine, Faculty of Medicine and Dentistry, University of Alberta, Edmonton, Alberta, Canada; 2 Department of Environmental and Occupational Health, School of Public Health, Drexel University, Philadelphia, Pennsylvania, USA; 3 Department of Public Health Sciences, School of Public Health, University of Alberta, Edmonton, Alberta, Canada; 4 School of Statistics and Management, Shanghai University of Finance and Economics, Shanghai, China; 5 Department of Pediatrics, Faculty of Medicine and Dentistry, University of Alberta, Edmonton, Alberta, Canada

## Abstract

**Background:**

Autism spectrum disorders (ASD) are associated with complications of pregnancy that implicate fetal hypoxia (FH); the excess of ASD in male gender is poorly understood. We tested the hypothesis that risk of ASD is related to fetal hypoxia and investigated whether this effect is greater among males.

**Methods:**

Provincial delivery records (PDR) identified the cohort of all 218,890 singleton live births in the province of Alberta, Canada, between 01-01-98 and 12-31-04. These were followed-up for ASD via ICD-9 diagnostic codes assigned by physician billing until 03-31-08. Maternal and obstetric risk factors, including FH determined from blood tests of acidity (pH), were extracted from PDR. The binary FH status was missing in approximately half of subjects. Assuming that characteristics of mothers and pregnancies would be correlated with FH, we used an Estimation-Maximization algorithm to estimate HF-ASD association, allowing for both missing-at-random (MAR) and specific not-missing-at-random (NMAR) mechanisms.

**Results:**

Data indicated that there was excess risk of ASD among males who were hypoxic at birth, not materially affected by adjustment for potential confounding due to birth year and socio-economic status: OR 1.13, 95%CI: 0.96, 1.33 (MAR assumption). Limiting analysis to full-term males, the adjusted OR under specific NMAR assumptions spanned 95%CI of 1.0 to 1.6.

**Conclusion:**

Our results are consistent with a weak effect of fetal hypoxia on risk of ASD among males. E-M algorithm is an efficient and flexible tool for modeling missing data in the studied setting.

## Background

The autism spectrum disorders (ASD) comprise a group of neurodevelopmental conditions that are associated with impaired verbal and non-verbal communication and social interaction, and restricted and repetitive patterns of behavior, which typically manifest before age of 3 years[1]. Although relatively rare[2-7], ASD can have a devastating effect on the quality of life of entire families and is associated with considerable societal economic burden[8]. There is some evidence that ASD prevalence is on the increase[9], although it remains unclear as to whether this increase can be completely accounted for by changes in case definition and improved ascertainment. While some genetic risk factors for ASD are known[10], the potential contribution of environmental factors has not been excluded and mediations involving gene-environment interactions would contribute to the observed genetic complexity in ASD. A recent review suggests that advanced parental age and fetal growth restriction place children at an elevated risk of developing ASD[11]. However, a recent meta-analysis concluded it is premature to implicate specific pregnancy complications in etiology of ASD[12]. Nonetheless, Kolevzon et al.[11], on the basis of epidemiological evidence, advanced a hypothesis that neonatal or fetal hypoxia are implicated in ASD, even though hypoxia was not measured in any of the reviewed studies. Chronically high maternal levels of dopamine have also been suggested by Previc[13] as the underlying cause of ASD, which would be expected to correlate with occurrence of fetal hypoxia. More recently Mueller and Bale[14] argued, based on studies of stress sensitivity in juvenile mice following prenatal hypoxic insults, that males are more susceptible to development of maladaptive stress responses associated with many neurodevelopmental disorders such as ASD. They suggested that this vulnerability is determined by sex-specific differences in placental physiology[14]. Excess of ASD among males is well-documented[15]. If fetal hypoxia were equally likely among boys and girls, but boys were more susceptible to its effect on ASD risk, this would contribute to explaining male predominance among ASD patients.

Parallel line of research suggests that neonatal encephalopathy caused by perinatal asphyxia/hypoxia in infants born at term is associated with neurological impairment later in life[16]. Therefore, it may well be that foetal hypoxia is simply an upstream cause of neonatal encephalopathy that then relates to risk of ASD. Indeed, the most recent review of the contribution of neonatal encephalopathy to infant neurological development concluded that even moderate post-asphyxia neonatal encephalopathy appears to contribute to cognitive and sensory-motor impairments, although heterogeneity in published results was noted[17].

In fact, one study of 239 children who survived neonatal encephalopathy reported that 4.2% were diagnosed with an ASD. This represents, after adjustment for confounders, about a 6-fold increase in ASD risk compared to ASD rates among 563 children who did not have neonatal encephalopathy (0.9%)[18]. The small sample size here led to render results of this report vulnerable to observing "significant" findings purely due to chance and the 95% confidence intervals of the relative risk estimate (2 to 17)[18] is extremely wide, casting doubts about the reproducibility of the finding[19].

None of the epidemiological studies conducted to date studied direct effects of hypoxia at birth on ASD. Therefore, we tested, in a population-based birth cohort, the hypothesis that risk of ASD is related to fetal hypoxia and investigated whether this effect is greater among males.

## Methods

### Record linkage

This cohort and analysis of association of perinatal risk factors with risk of ASD are described elsewhere, without consideration of fetal hypoxia[20], defined as fetal scalp pH < 7.25, or umbilical artery pH < 7.20, or venous pH < 7.28. Delivery records held by the Alberta Perinatal Health Program (APHP) identified the cohort of singleton live births in the province of Alberta, Canada, between January 1, 1998 and December 31, 2004. The APHP provided information regarding relevant ante- and peri-natal risk factors. Information on the risk factors was collected on admission to hospital for delivery, as part of routine clinical care. They are considered to be accurate and any internal inconsistency in the records is scrutinized by APHP; if apparent error in the delivery records cannot be resolved, APHP records a missing value for a given variable. The unique Personal Health Number (PHN) of mother and child along with child's gender and date of birth in APHP file were used to follow-up the cohort through records held by the Alberta Health and Wellness (AHW). In the universal healthcare insurance system of Alberta, all residents are served by physicians who bill the government for their services, linked to specific diagnostic codes, International Classification of Diseases 9^th ^edition (ICD-9). Children matched in APHP file to AHW records were followed-up by the investigators until March 31, 2008 for (a) ICD-9 codes indicating ASD (299.0 or 299.8) listed with the physician billing record, (b) child's residency and mortality (in a given fiscal year ending on March 31) and (c) mothers' socioeconomic status. Outcome was defined using most liberal definition from our previous work: at least one ASD claim by any physician because such case definition showed good sensitivity and specificity and did not affect associations between studied risk factors and ASD[20]. Such case definition for ASD is analogous to the one used in another, very similar Canadian study, where it was shown to have acceptable sensitivity and specificity relative to gold standard diagnostic assessment in a specialty clinic[21]. Study protocol was approved by the University of Alberta Health Ethics Research Board and the custodians of the data.

### Statistics

**Figure 1:**
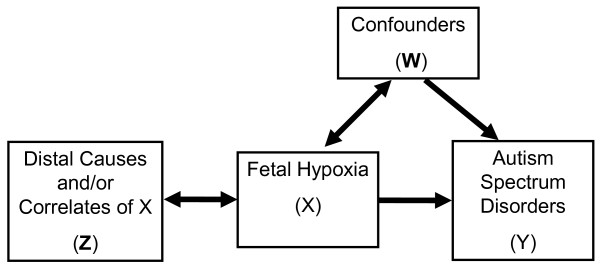
**The hypothesized relationship between birth complications and maternal factors (Z), fetal hypoxia (X), sex, socio-economic class and birth cohort (W), and autism spectrum disorders (Y) (when stratified on sex, W is just socio-economic class and birth cohort); bold font denotes a vector**.

Causal model considered in this paper is shown in Figure 1. Our hypothesis is that ASD (Y) is caused by fetal hypoxia (X). Birth year and socio-economic status (W) are treated as confounders (as is fetal gender unless the effect modification by gender is tested), because there is little reason to suspect that such broad proxies for trends in health (social-economic status) and chance of being diagnosed (birth year) would only act as antecedents of fetal hypoxia without also being associated with risk of ASD. We make a very strong assumption that all other covariates (Z) are assumed to be correlated with ASD through fetal hypoxia, but relax it when we test the notion that prematurity can also have a direct effect on the risk of ASD. Our justification for this approach is that we focus on testing a specific well-justified hypothesis. In most of the analyses, unless otherwise indicated, covariates Z include maternal age (≤ 25, 25- ≤ 30, 30- ≤ 35, >35 years), maternal weight (>91 kg or < 45 kg), maternal height < 152 cm, pre-pregnancy diabetes, gestational diabetes, bleeding (< 20 and/or > 20 weeks), cigarette smoking (any), poor weight gain (26-36 weeks < 0.5 kg/week), parity (0, 1, 2, 3, ≥4), pre-eclampsia, presentation (cephalic or breech/shoulder), type of labor (spontaneous, induced, no labor/C-section), gestational age (< 37,≥37 weeks), birth weight (< 2.5, 2.5-4.5, > 4.5 kg), Apgar at 1 and 5 min (< 7). A logistic regression model was fitted to data relating odds of ASD to fetal hypoxia, to estimate odds ratios (ORs) and associated 95% confidence intervals (CIs).

**Figure 2:**
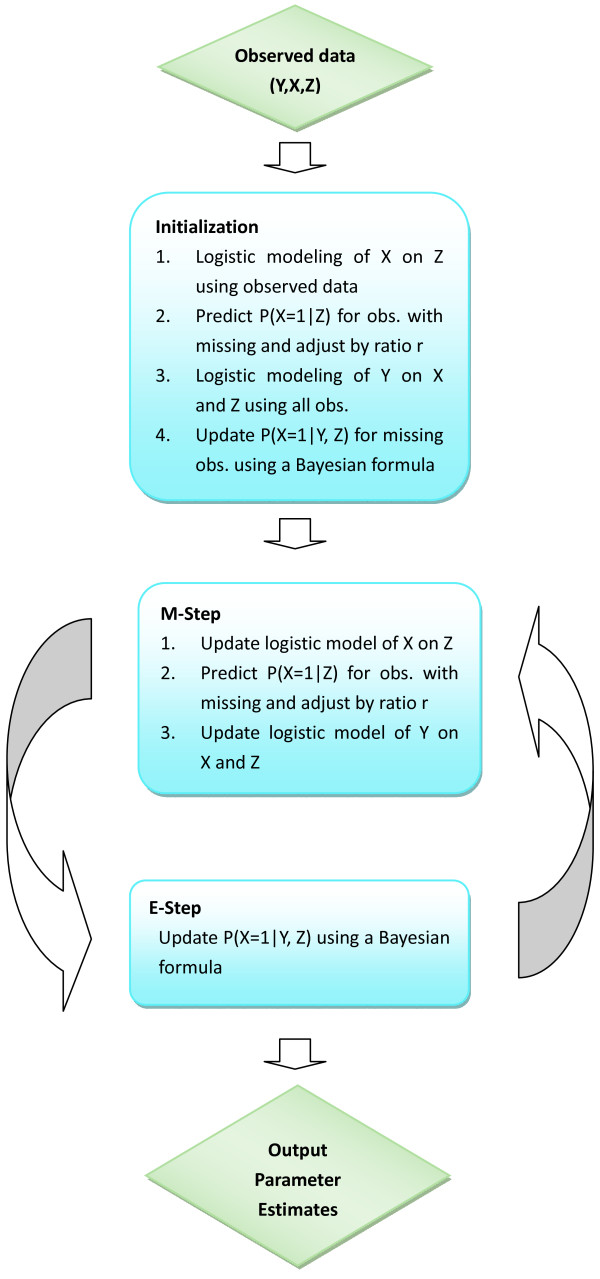
**Flow chart of EM Algorithm (refer to Appendices for mathematical details and implementation)**.

Fetal hypoxia status, the binary exposure variable of interest (X), was missing in approximately one half of our study subjects. Based on the notion that some variables (Zs) including characteristics of mothers and pregnancies would be correlated with the exposure X, we used an EM algorithm[22] to tackle the estimation problem of the association between fetal hypoxia (X) and ASD (Y), in the presence of missing values in X. The basic idea of the EM algorithm is to use subjects' characteristics Zs and the outcome Y to model X using the subjects with observed values of X. With the model for X with Zs and Y, we can weigh the two possibilities of the missing value of X, X = 1 and X = 0, in the likelihood function for maximum-likelihood statistical inference on parameters. Since the model for X with Zs and Y contains the parameters of interest, the weighted likelihood function can be iteratively maximized: estimating the weights using the current parameter estimates; and constructing the weighted likelihood function, which can then be maximized to update the parameter estimates. We initially make an assumption that, given Z and Y, missingness of X occurs at random: the missing-at-random (MAR) assumption, which assumes random missing conditioned on Z and Y. Under the MAR conditioned on Z and Y, using those who have X for modeling X with Z and Y is valid. It is reasonable to doubt the validity of MAR assumption in this situation, if we consider the possibility that the likelihood of X = 1 is systematically higher for a given Z among those that were tested for fetal hypoxia. For example, clinicians can consider factors other than those measured as Zs in ordering test for fetal hypoxia, such that their suspicion of a positive result, over and beyond what Zs suggest, is elevated before the test is performed. If this were the case, then missingness in X is not at random (not-missing-at-random, NMAR). To consider the NMAR case, we introduce (0 ≤ *r *≤ 1), the ratio of expected probabilities of having hypoxia in X missing group and non-missing group for a given set of Zs. Given information from Zs, setting = 1 means we assume MAR. Any case with < 1 amounts to postulating a special case of NMAR. There is no data on how much smaller than 1 *r *should be in our particular setting. Such values can arise from separate experiments (not possible in our situation), or sensible guesses of values of *r *can be made for the purposes of sensitivity analysis (route we followed); yet another approach is to follow fully Bayesian paradigm and place a prior on *r*. It is not possible to optimize *r *from our data using EM algorithm. We considered in sensitivity analyses a range of *r *spanning values consistent with excellent (*r *= 0.1) to moderate (*r *= 0.7) additional clinical judgment indicating the likelihood of positive test, over and beyond Zs. Note that *r *= 0.5 suggests that those not tested are only at 50% risk of hypoxia (given Zs) compared to those who were tested: this is consistent with the notion of predictive ability of the clinical judgment, beyond Zs, that the hypoxia test is positive. As we shall see below (Table 1), intensive testing for hypoxia is associated with a factor of 2-5 increase in detecting hypoxia, which gives strong evidence that testing is ordered only on strong suspicion of hypoxia and that *r *is in the range of 0.5 or smaller. All EM algorithms used in this paper converged within 250 iterations. Mathematical details are given in the Additional File 1 and sketch of SAS (SAS Institute, Cary, NC) code required to implement the method is given in Additional File 2. Schematic presentation of EM algorithm we implemented is given in Figure 2.

**Table 1:** Ascertainment of Fetal Hypoxia^1^ Among Neonates

Test(s)	Subjects tested	All tests positive		At least one test positive	
		Number of subjects	%	Number of subjects	%

*All three tests*	26	1	4	12	46

*Two tests*					

Fetal scalp and umbilical arterial blood pH	178	27	15	77	43

Umbilical arterial and venous pH	41,245	3,583	9	8,866	21

Fetal scalp and umbilical venous blood pH	4	0	0	0	0

*One test*					

Fetal scalp pH	47	16	34	16	34

Umbilical arterial blood pH	75,775	7,727	10	7,727	10

Venous umbilical blood pH	3,561	385	11	385	11

*Any of the three tests*	120,836	11,739	10	17,083	14

1: fetal scalp pH < 7.25, umbilical artery pH < 7.20, or umbilical venous pH < 7.28

## Results

There were 273,343 singleton live births in Alberta according to APHP records between 1998 and 2004, among whom 25,970 children could not be identified by AHW and 28,421 either died, or lost residence during follow-up; a further 62 had missing gender, leaving 218,890 children for analysis. There were no systematic differences on observable data among births that were included and excluded (details not shown). In 48% (105,636/218,890) of births, fetal hypoxia tests via blood pH were performed. In a minority of births multiple tests were performed (Table 1). A total of 17,083 tests (14%) of the 120,836 that were preformed satisfied our definition of fetal hypoxia: positive on at least one of the (multiple) tests. Umbilical arterial blood pH was the most common test used either on its own (n = 75,775) or in combination with venous blood pH (n = 41,245). As expected, there was some disagreement among tests on the same neonate. However, overall prevalence of fetal hypoxia among those tested appeared to be similar whether all tests were required to be congruent (10%) or only one had to be positive (14%). Measurement of fetal scalp pH, although relatively infrequent, appears to be associated with elevated likelihood of the overall positive test result, perhaps because not of the inherent superior sensitivity of the test, but because it is administered only when there is considerable suspicion that the fetus may be hypoxic. There were 200,557 full-terms births in the cohort and gestational age at birth was missing for 444 neonates. 1,011 of full-terms neonates were diagnosed with ASD during the follow-up.

Table 2 summarizes data on ASD and measures of fetal hypoxia as observed for births of all gestational ages. Among the subgroup of 17,083 infants classified as hypoxic, 113 were diagnosed with ASD during the follow-up (0.66%). The observed rate of ASD was slightly lower among 88,553 infants who tested negative for fetal hypoxia (530 ASD cases, 0.60%) and much lower among subjects 113,254 whose fetal hypoxia status was unknown, that is mostly not tested (495 ASD cases, 0.44%). The observed rate of ASD was the highest among males who were hypoxic at birth (100 ASD cases, 1.10%). Males who were tested and did not appear to be hypoxic at birth had a slightly lower observed rate of ASD (452 ASD cases, 0.99%), but males who were not tested for hypoxia certainly had a reduced observed rate of ASD compared to those males who were tested (400 ASD cases, 0.70%). The observed rates of ASD among female children did not vary with measures of fetal hypoxia (0.16% among hypoxic vs. 0.18% among non-hypoxic) and was much lower than among males (1.10% among hypoxic vs. 0.99% among non-hypoxic).

**Table 2:** Summary of Data on Reported Fetal Hypoxia and Diagnosis of Autism Spectrum Disorders in the Same Child (All Gestational Ages at Birth)^1^

Child's Gender	Autism Spectrum Disorders	Fetal Hypoxia			Total
		**Present^2^**	**Absent^4^**	**Unknown**	

Both	Present N	113	530	495	1,138
	**%**^3^	**0.66**	**0.60**	**0.44**	**0.52**
		(0.06)	(0.03)	(0.02)	(0.02)

	**Total **N	17,083	88,553	113,254	218,890

Male	Present N	100	452	400	952
	**%**^3^	**1.10**	**0.99**	**0.70**	**0.85**
		(0.11)	(0.05)	(0.03)	(0.03)

	**Total **N	9,109	45,594	57,257	111,960

Female	Present N	13	78	95	186
	**%**^3^	**0.16**	**0.18**	**0.17**	**0.17**
		(0.04)	(0.02)	(0.02)	(0.01)

	**Total **N	7,974	42,959	55,997	106,930

1: singletons born in Alberta 1998-2004 and followed up till March 31, 2008

2: at least one of the three tests for fetal hypoxia is positive, i.e. fetal scalp pH < 7.25, or umbilical artery pH < 7.20, or venous pH < 7.28

3: percentage of exposed to different states of fetal hypoxia (with standard errors)

4: excludes subjects with base pH, i.e. this reference category has pH in normal physiological range

Among full-term births the pattern of observed rates is similar to that seem among all births in Table 2. The observed ASD rate among full-terms births was higher among those who tested positive for hypoxia (0.62%, 96/15,557) compared to those who tested negative for hypoxia (0.58%, 557/95,352). The test for hypoxia was not performed among 105,205 members of this sub-cohort who had an even lower observed rate of ASD (454, 0.43% affected). 480 of 49,165 full-term male births with data on hypoxia were diagnosed with ASD during the follow-up (observed rate 0.98%). Among 8,286 males hypoxic at birth, the observed rate of ASD was 1.03% (85 cases), but among males not hypoxic at birth the observed rate of ASD was slightly lower: 0.97% (395 cases). In this male sub-cohort, when information on hypoxia was missing, the observed rate of ASD was even lower 0.69% (364 cases). 77 of 46,187 full-term female births with data on hypoxia were diagnosed with ASD during the follow-up (observed rate 0.17%). Among 7,271 females hypoxic at birth, the observed rate of ASD was 0.15% (11 cases), but among females not hypoxic at birth the observed rate of ASD was higher: 0.17% (66 cases). In this female sub-cohort, when information on hypoxia was missing, the observed rate of ASD was the same as among girls who tested as not hypoxic at birth: 0.17% (90 cases).

We will next examine ASD rate modeled using E-M algorithm among births of all gestational ages. Table 3 (top) presents results from E-M algorithm that modeled the presence of fetal hypoxia for 113,254 out of 218,890 subjects and then estimated odds ratios for the association of ASD with fetal hypoxia with and without adjustment for suspected confounders (Ws) and with and without stratification on child's gender. In stratified analyses, strata-specific modeling of missing exposures was performed using values of Zs specific to each gender. There is a clear suggestion of a small excess risk of ASD only among males who were hypoxic at birth: unadjusted OR 1.14, 95%CI: 0.97, 1.34. This result is not materially affected by adjustment for potential confounding due to birth year and socio-economic status: adjusted OR 1.13, 95%CI: 0.96, 1.33. As expected from descriptive analyses (Table 2), there is no indication that ASD risk among females is associated with positive test for fetal hypoxia. The formal test of the (multiplicative) interaction of male gender and hypoxia on the risk of ASD did not indicate that males had a higher degree of association between hypoxia and ASD than females: *P *= 0.68 for interaction OR of 0.91 (details of the model not shown). Given uncertain role that prematurity plays in ASD etiology[23], we could not justify treating it a sole antecedent of hypoxia and not a risk factor on its own. To control for possible confounding effect of prematurity we repeated the analysis on full-term births only (Table 3, bottom). The results are largely consistent with those obtained on full cohort, but suffer from loss of precision. The observation provides some further justification in not treating Z as confounders in the bulk of our analyses.

**Table 3:** Association of Fetal Hypoxia at Birth^1^ and Diagnosis of Autism Spectrum Disorders^2^: Results of Logistic Regression Fitted with EM Algorithm for Handling Unknown Exposures That are Modeled From Maternal and Birth Characteristics Under MAR Assumption (r = 1) Stratified on Child's Gender

All gestational ages at birth						
	**Unadjusted**			**Adjusted**		

Child's Gender	Both	Male	Female	Both^3^	Male^4^	Female^4^

**Odds ratio estimate**	1.15	1.14	0.93	1.11	1.13	0.94

**95% confidence interval**	0.98, 1.33	0.97, 1.34	0.62, 1.40	0.95, 1.29	0.96, 1.33	0.62, 1.41

**P-value**	0.08	0.11	0.74	0.19	0.14	0.76

**Full-term births only**						

	**Unadjusted**			**Adjusted**		

Child's Gender	Both	Male	Female	Both^3^	Male^4^	Female^4^

**Odds ratio estimate**	1.11	1.10	0.92	1.07	1.09	0.93

**95% confidence interval**	0.94, 1.31	0.93, 1.31	0.60, 1.42	0.91, 1.34	0.91, 1.29	0.60, 1.56

**P-value**	0.21	0.23	0.71	0.42	0.35	0.74

1: at least one of the three tests for fetal hypoxia is positive, i.e. fetal scalp pH < 7.25, or umbilical artery pH < 7.20, or venous pH < 7.28

2: singletons born in Alberta 1998-2004 and followed up till March 31, 2008

3: adjusted for gender, birth year and socio-economic status

4: adjusted for birth year and socio-economic status

Lastly, we investigated the impact of specific NMAR assumption on our results in a sub-set of full-term male infants where the conservative analyses under the MAR assumption indicated a possible positive association (Table 4). There was also evidence that missingness of data on hypoxia was associated with risk of ASD in males, but not in females. Under the realistic NMAR assumption (*r *from 0.1 to 0.5), it appears that point estimate of the effect (OR) of hypoxia on risk of ASD among males who were born at full-term increases to 1.2-1.3 with 95%CIs that lie approximately between 1.0 and 1.6. Even if deviation from the MAR assumption was weak (*r *= 0.7), the resulting change in inference is dramatic: 95%CIs widen somewhat and shift from 0.91-1.29 (Table 3) to 0.99-1.46 (Table 4), while the point estimate increases from 1.09 (Table 3) to 1.20 (Table 4).

**Table 4:** Association of Fetal Hypoxia at Birth1 and Diagnosis of Autism Spectrum Disorders2: Results of Logistic Regression Fitted with EM Algorithm for Handling Unknown Exposures That are Modeled From Maternal and Birth Characteristics Under Specific NMAR Assumption Among Full-Term Male Births

Deviation from MAR^3^	Strong	Strong	Moderate	Weak
*r*	*0.1*	*0.2*	*0.5*	*0.7*

**Odds ratio estimate**	1.28	1.28	1.24	1.20

**95% confidence interval**	1.03, 1.60	1.03, 1.58	1.01, 1.52	0.99, 1.46

**P-value**	0.03	0.03	0.04	0.06

1: at least one of the three tests for fetal hypoxia is positive, i.e. fetal scalp pH < 7.25, or umbilical artery pH < 7.20, or venous pH < 7.28

2: singletons born in Alberta 1998-2004 and followed up till March 31, 2008, effects adjusted for birth year and socio-economic status

3: contribution of clinical judgment to prescribing the test beyond Z; MAR = missing at random, *r *= 1, see Table 3

## Discussion

There are many difficulties with these data beyond missing-ness: both exposures and outcomes are suspected of being misclassified, which would bias apparent OR estimates, most likely towards the null if both misclassification mechanisms are non-differential. Covariates from perinatal database are known to be recorded accurately [Ms N. Bott of APHP, personal communications] because they use data essential to medical care, not just billing, even though some information (such as maternal smoking[24] used to model risk of fetal hypoxia) is known to be collected with error. Of course error in variables can bias our results, but we lack information on the extent and direction of such bias. Our analysis is also limited by small number of girls, so that our failure to detect an association among them may simply be due to low power. Short follow-up for some members of the cohort born in 2004 may have introduced error due to incomplete ASD ascertainment, since they were be only 4 at the end of follow-up, but our previous results suggest that in this population peak age of diagnosis was between ages of 3 and 4 [20], therefore our data cannot address this potential limitation. Nonetheless, our data are valuable because they enable the first test of association of fetal hypoxia with risk of ASD in a population-based birth cohort. Innovative statistical methodology that we used to meet challenged posed by the data should prove to be helpful to researchers facing similar complications.

We observed that males who were tested for foetal hypoxia were more likely to develop ASD. This supports the generally establish contention that ASD patients tend to have complicated pregnancies[11], which often involve, upon suspicion, testing for foetal hypoxia. Presence of hypoxia itself, under the causal models that we considered, appears to only affect males. This finding is consistent with our *a priori *specified hypothesis about greater sensitivity of males to hypoxia-induced insults during gestation.

We conducted most of our analyses under a strong assumption that the majority of observed pregnancy and birth characteristics only affected risk of ASD through fetal hypoxia. In doing so, we reflected on discussion by Newschaffer and Cole[25] of testing alternative causal models in studies of perinatal risk factors in ASD. Since our entire analysis is predicated on apparent association of hypoxia with pregnancy and birth characteristics (Z), we can reject the "etiologic heterogeneity" model[25]: if hypoxic status was independent of Z we would not have been able to implement analysis that relies on EM-algorithm. We tested *a prior *justified effect modification by gender and report non-multiplicative interaction on the basis of stratified analysis. Then we have to consider "epiphenomena" models [25]. "Epiphenomena model 2" is in fact represented by Figure 1 and was estimated here directly. With respect to "epiphenomena model 1", it is currently unreasonable to suspect that ASD causes fetal hypoxia, since that would postulate a fetus that is both destined to have ASD and able to affects its own supply of oxygen. This leaves the possibility that "shared risk factor" model[25] applies and there may be confounding by direct effect of elements of Z on risk of ASD if model in Figure 1 is fitted to the data. We considered confounding by prematurity: in a stratum of full-term infants for who we had sufficient sample size to obtain meaningful estimates (92% of all subjects) we found little evidence that prematurity induced association between hypoxia and ASD. None of the other elements of Z appeared to be likely candidates for examining the direct effect on risk of ASD. Of course given how little we know about causes of ASD, it is possible that we failed to postulate and test true causal model. If that is the case, then our estimates may in fact be biased by latent confounding or effect modification, which is of course true of most observational research into subject matters, such as ASD, where uncertainty abounds about what the actual causes may be.

Missing values are common in public health research. Understanding the missing mechanism is the key for a proper missing data analysis. Relaxing unrealistic MAR assumptions changed the point estimate of the disease-exposure odds ratio and sifted confidence intervals (P-values) towards the region conventionally thought of as "significant". Thus, the use of EM-algorithm for dealing with the special missing value problem in this project in conjunction with sensitivity analysis using the specified NMAR assumption altered statistical inference and was therefore useful.

## Conclusions

Our results are consistent with a weak effect of fetal hypoxia on risk of ASD that is confined to males. It may well be that some latent indication or an unknown correlated of testing for hypoxia at birth are responsible for the increased risk of ASD among males and therefore investigation of role of hypoxia at birth in ASD remains a fruitful area for research.

## Competing interests

The authors declare that they have no competing interests.

## Authors' contributions

All authors credited with this work made substantial intellectual contribution to the submitted manuscript. IB led overall project; the current analysis address the hypothesis he developed and attempted to test. IB, SF and LZ designed the study, oversaw data acquisition, contributed to analysis and interpretation of the data and co-wrote the manuscript. XW and YY devised and implemented data analysis via E-M algorithm, they led the preparation of technical appendices and made substantial contribution to writing the main body of the manuscript. All authors reviewed and approved the submitted version of the manuscript.

## Pre-publication history

The pre-publication history for this paper can be accessed here:

http://www.biomedcentral.com/1471-2288/11/2/prepub

## Supplementary Material

Additional file 1 **EM Algorithm with either MAR or NMAR assumptions**. Mathematical details of EM algorithm implemented in this article. Click here for file

Additional file 2 **Implementation of EM Algorithm for missing values in SAS software**. SAS code that was used to implement EM algorithm presented in this article with annotation that allow adapting it to similar applications. Click here for file
