# Supplementary material for: Autism spectrum disorders and fetal hypoxia in a population-based cohort: Accounting for missing exposures via Estimation-Maximization algorithm
Source: BMC Med Res Methodol. 2011 Jan 5;11:2. doi: 10.1186/1471-2288-11-2 (PMC3024997; doi:10.1186/1471-2288-11-2)
Supplement: Additional file 1 — EM Algorithm with either MAR or NMAR assumptions. Mathematical details of EM algorithm implemented in this article. [file 1471-2288-11-2-S1.DOC]

**Appendix 1: EM Algorithm with either MAR or NMAR assumptions**

**Assumptions**

Let $\left\{(Y_{i},X_{i},Z_{i},M_{i}), i=1,...,n\right\}$be data of a sample of size $n$, where $M_{i}=0$ and $1$ indicating $X_{i}$ (hypoxia) is observed ($X_{i}=X_{i}^{obs}$) and missing ($X_{i}=X_{i}^{mis}$), respectively. We assume that $X$can be explained by $Z$(covariates), and that $Y$(ASD outcome) can be explained by$X$ and $Z$, using the following logistic model and mixture logistic model

$\Pr(Y_{i}|X_{i},Z_{i})=\frac{1}{1+\exp(-\beta_{0}-(X_{i},Z_{i})^{T}\beta)}$ (1)

$\Pr(X_{i}|Z_{i})=\delta(M_{i})\frac{1}{1+\exp(-\gamma_{0}-Z_{i}^{T}\gamma)},$ (2)

where $\delta(M_{i})$= $r$ and 1 for $M_{i}=$1 and 0, respectively. Model (2) implies the following assumption on the missing mechanism of X

$E(X_{i}|Z_{i},M_{i}=1)=rE(X_{i}|Z_{i},M_{i}=0)$, (3)

where $r$ $(0\leq r\leq1)$is a ratio of probabilities of having hypoxia between the missing group and the non-missing group. Given Z, setting $r$=1 means we assume the missing is at random (MAR); any case with $r$ < 1 amounts to say the missing is not at random (MNAR).

By$\Pr(Y_{i},X_{i}|Z_{i})=\Pr(Y_{i}|X_{i},Z_{i})\Pr(X_{i}|Z_{i})$, the conditional log-likelihood given $Z$ can be written as

$$L(\beta_{0},\beta,\gamma_{0},\gamma,X^{mis})=\sum_{\{i:M_{i}=0\}} \{\mathrm{logPr}(Y_{i}|X_{i}^{obs},Z_{i})+\log(X_{i}^{obs}|Z_{i})\}$$

$$+\sum_{\{i:M_{i}=1\}} \{\mathrm{logPr}(Y_{i}|X_{i}^{mis},Z_{i})+\log(X_{i}^{mis}|Z_{i})\}$$

$$=L_{1}(\beta_{0},\beta,X^{mis})+L_{2}(\gamma_{0},\gamma,X^{mis})$$

where,

$$L(\beta_{0},\beta,X^{mis})=\sum_{\{i:M_{i}=0\}} \mathrm{logPr}(Y_{i}|X_{i}^{obs},Z_{i})+\sum_{\{i:M_{i}=1\}} \mathrm{logPr}(Y_{i}|X_{i}^{mis},Z_{i})$$

$$L(\gamma_{0},\gamma,X^{mis})=\sum_{\{i:M_{i}=0\}} \mathrm{logPr}(X_{i}^{obs}|Z_{i})+\sum_{\{i:M_{i}=1\}} \mathrm{logPr}(X_{i}^{mis}|Z_{i})$$

are conditional log-likelihood of model (1) and (2), respectively.

**E-step and M-step:**

The EM algorithm is an iterative procedure to maximize the conditional log-likelihood, in which each iteration has an E-step and an M-step. For each $i$ with $M_{i}=1$, let $\pi_{i}=\Pr(X_{i}^{mis}=1|Y_{i},Z_{i})$. The expectation of the conditional log-likelihood can be written as

$$E_{X^{mis}}L(\beta_{0},\beta,\gamma_{0},\gamma,X^{mis})$$

$$=\sum_{\{i:M_{i}=0\}} \mathrm{logPr}(Y_{i}|X_{i}^{obs},Z_{i})$$

$$+\sum_{\{i:M_{i}=1\}} \{\pi_{i}\mathrm{logPr}(Y_{i}|X_{i}^{mis}=1,Z_{i})+(1-\pi_{i})\mathrm{logPr}(Y_{i}|X_{i}^{mis}=0,Z_{i})\}$$

$$=\sum_{\{i:M_{i}=0\}} \mathrm{logPr}(X_{i}^{obs}|Z_{i})$$

$$+\sum_{\{i:M_{i}=1\}} \{\pi_{i}\mathrm{logPr}(X_{i}^{mis}=1|Z_{i})+(1-\pi_{i})\mathrm{logPr}(X_{i}^{mis}=0|Z_{i})\}$$

The key point in E-step is how to estimate $\pi_{i}$.

If $\pi_{i}$ can be predicted properly, then the M-step can be done separately. The first term of $E_{X^{mis}}L(\beta_{0},\beta,\gamma_{0},\gamma,X^{mis})$ is a weighted logistic regression, from which we can get estimates of $\beta$ coefficients; and the second term is also a weighted logistic regression form which we get estimates of $\gamma$coefficients.

**Predicting** $\pi_{i}$

The key is predicting $\pi_{i}$ using information form $Z_{i}$ and $Y_{i}$, along with current estimates $\beta_{_{0}}^{C}$, $\beta^{C}$,$\gamma_{0}^{C}$ and $\gamma^{C}$of the parameters. Here, we suggest using a Bayesian formula as follows.

$$\pi_{i}=\Pr(X_{i}=1|Y_{i}=0,Z_{i})$$

$$=\frac{\Pr(X_{i}=1|Z_{i})\Pr(Y_{i}=0|X_{i}=1,Z_{i})}{\Pr(X_{i}=0|Z_{i})\Pr(Y_{i}=0|X_{i}=0,Z_{i})+\Pr(X_{i}=1|Z_{i})\Pr(Y_{i}=0|X_{i}=1,Z_{i})}$$

$$\pi_{i}=\Pr(X_{i}=1|Y_{i}=1,Z_{i})$$

$=\frac{\Pr(X_{i}=1|Z_{i})\Pr(Y_{i}=1|X_{i}=1,Z_{i})}{\Pr(X_{i}=0|Z_{i})\Pr(Y_{i}=1|X_{i}=0,Z_{i})+\Pr(X_{i}=1|Z_{i})\Pr(Y_{i}=1|X_{i}=1,Z_{i})}$,

where $\Pr(X_{i}|Z_{i})$ can be estimated in model (2) using current $\gamma$ coefficients; and $\Pr(Y_{i}|X_{i},Z_{i})$ can be estimated in model (1) using current $\beta$ coefficients

**Implementation**

In our data analysis, the relationship between $X$ and $Z$ in model (2) is specified as

$$\Pr(X_{i}|\bar{Z}_{i})=\delta(M_{i})\frac{1}{1+\exp(-\gamma_{0}-\bar{Z}_{i}^{T}\gamma)}$$

where $\bar{Z}$ includes all features of $Z$ but birth-cohort, gender and socio-economic status; while model (1) is specified as either unadjusted (i.e. *X* is the only covariate) or adjusted (i.e. X, birth-cohort, gender, socio-economic status are covariates) models. The iterative algorithm was coded in SAS and the process of iteration continued untill values of the conditional maximum likelihood converged.

**Reference**

McLachlan G. J. and Krishnan T. The EM Algorithm and Extensions, JOHN WILEY & SON, INC. 1997
