# Supplementary material for: Autism spectrum disorders and fetal hypoxia in a population-based cohort: Accounting for missing exposures via Estimation-Maximization algorithm
Source: BMC Med Res Methodol. 2011 Jan 5;11:2. doi: 10.1186/1471-2288-11-2 (PMC3024997; doi:10.1186/1471-2288-11-2)
Supplement: Additional file 2 — Implementation of EM Algorithm for missing values in SAS software. SAS code that was used to implement EM algorithm presented in this article with annotation that allow adapting it to similar applications. [file 1471-2288-11-2-S2.DOCX]

/***************************************************************************************/

/* Appendix 2 : Implementation of E-M Algorithm for missing values in SAS */

/***************************************************************************************/

/*adjustment for special case of NMAR are highlighted in yellow, illustrated for r=0.5*/

/**** Initial 1. Logistic regression of exposure X give Z’s ****/

**proc** **logistic** data=data descending;

model X=z1 z2 z3 /*as many Z's as required*/ ;

output out=pX PREDICTED=px;

**run**;

/**** Initial 2. data adjustment: X_i^{mis}=0/1 with weight p_i/(1-p_i) ****/

**data** pX(drop=i);

set pX;

if X=**.** then px=px*0.5 /*adjust the probability of px in missing group for NMAR r=0.5*/;

**run;**

**data** pX(drop=i);

set pX;

if X=**.** then do;

do i=**0** to **1**;

IX=i;

if i=**0** then weight=**1**-px;

else weight=px;

output;

end;

end;

else do;

IX=X;

weight=**1**;

output;

end;

**run**;

/**** Initial 3. Estimate Pr(Y_i|Z_i,X_i) ****/

**proc** **logistic** data=pX descending;

class IX;

model y=IX;

weight weight;

output out=pX PREDICTED=py;

**run**;

/**** Initial 4. Initial weights Pr(X_i|Z_i,Y_i) ****/

**data** pX(drop=_a);

set pX;

if X=**.** then do;

if iX=**0** and y=**1** then do;

_a=py;

weight=(**1**-px)*_a/(px*py+ (**1**-px)*_a);

end;

else if iX=**1** and y=**1** then weight=px*py/(px*py+ (**1**-px)*_a);

else if iX=**0** and y=**0** then do;

_a=py;

weight=(**1**-px)*(**1**-_a)/(px*(**1**-py)+ (**1**-px)*(**1**-_a));

end;

else if iX=**1** and y=**0** then weight=px*(**1**-py)/(px*(**1**-py)+ (**1**-px)*(**1**-_a));

else weight=**1**;

end;

retain _a;

**run**;

**Data** pX ;

set pX(drop=px _LEVEL_ _LEVEL_2 py);

**run**;

/***************************************************************************************/

/**************** iterative process of EM algorithm ************************/

/***************************************************************************************/

**%macro** logit_imputation(first=**1**, last=**5000**);

%local it;

%do it=&first %to &last;

/**** iterative step 1 (weighted logistic regression for predicting Pr(X_i|Z_i)***/

ods output FitStatistics=logforL; /* ods= output deliver system*/

proc logistic data=pX descending;

model IX=z1 z2 z3 /*as many Z's as required*/;

weight weight ;

output out=pX PREDICTED=px;

run;

ods output close;

/**** iterative step 2 (weighted logistic regression for prediction Pr(Y_i|X_i,Z_i)***/

ods output ParameterEstimates=est_p;

proc logistic data=pX descending;

class IX ;

model y=IX;

weight weight;

output out=pX PREDICTED=py;

run;

ods output close;

/**** iterative step 3 (adjust weights using Bayesian inference)***/

**data** pX(drop=i);

set pX;

if X=**.** then px=px*0.5 /* adjust the probability of px in missing group */;

**run;**

data pX(drop=_a);

set pX;

if X=**.** then do;

if iX=**0** and y=**1** then do;

_a=py;

weight=(**1**-px)*_a/(px*py+ (**1**-px)*_a);

end;

else if iX=**1** and y=**1** then weight=px*py/(px*py+ (**1**-px)*_a);

else if iX=**0** and y=**0** then do;

_a=py;

weight=(**1**-px)*(**1**-_a)/(px*(**1**-py)+ (**1**-px)*(**1**-_a));

end;

else if iX=**1** and y=**0** then weight=px*(**1**-py)/(px*(**1**-py)+ (**1**-px)*(**1**-_a));

else weight=**1**;

end;

retain _a;

run;

/**** iterative step 5 (drop px _LEVEL_ _LEVEL_2 py)***/

Data pX ;

set pX(drop=px _LEVEL_ _LEVEL_2 py);

run;

proc append base=logall data=logforl force; run;

proc append base=pvalue data=est_p force; run;

%end;

**%mend** logit_imputation;

%***logit_imputation***(first=**1**,last=**250**) *for 250 itreations;

**data** outdata.result1_z2x_250; set logall; **run**;

**data** outdata.result1_x2y_250; set pvalue; **run**;
